# New Neurons in the Postnatal Olfactory System: Functions in the Healthy and Regenerating Brain

**DOI:** 10.3390/brainsci15060597

**Published:** 2025-06-02

**Authors:** Jordan D. Gregory, Tenzin Kunkhyen, Sean C. Sweat, Jane S. Huang, Taryn R. Brechbill, Claire E. J. Cheetham

**Affiliations:** 1 Department of Neurobiology, University of Pittsburgh, Pittsburgh, PA 15261, USA; 2 Center for Neuroscience, University of Pittsburgh, Pittsburgh, PA 15261, USA

**Keywords:** postnatal neurogenesis, olfactory epithelium, subventricular zone, learning, regeneration

## Abstract

The rodent olfactory system is unique in harboring two distinct postnatal neurogenic niches, the olfactory epithelium and the subventricular zone. This results in the ongoing generation of both olfactory sensory neurons (OSNs), which provide odor input to the brain, and multiple molecularly distinct populations of GABAergic interneurons that modulate both input to and output from the olfactory bulb, continuing throughout life for some neuronal types. Here, we review the roles played by these postnatally generated neurons in olfactory processing, plasticity and regeneration. We identify specific roles for individual types of postnatally generated neurons, as well as identifying overarching principles that span multiple neuronal types.

## 1. Introduction

**Figure 1:**
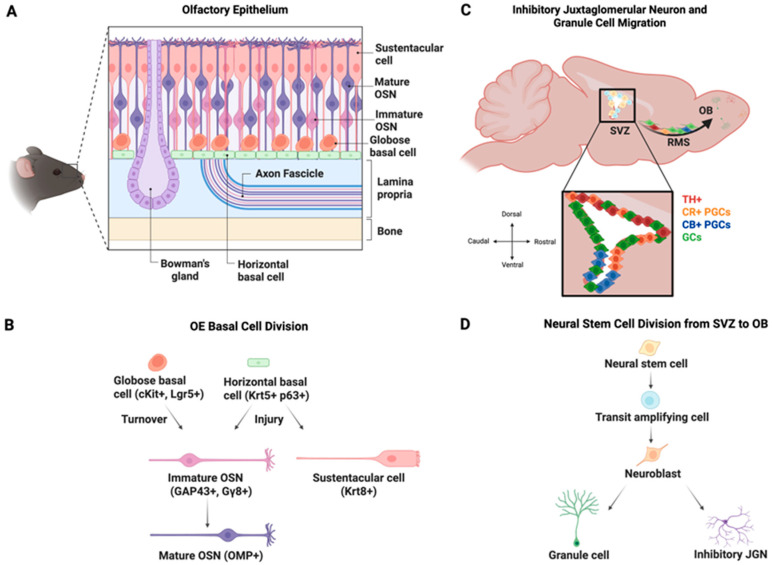
Overview of postnatal neurogenesis in the rodent olfactory epithelium (OE) and subventricular zone (SVZ). (**A**) Pseudostratified organization of the OE showing basal cells, immature and mature olfactory sensory neurons (OSNs). An axon fascicle projecting through the lamina propria to the OB is also shown. (**B**) In the healthy OE, globose basal cells (GBCs) generate immature OSNs, which migrate apically as they mature. Horizontal basal cells (HBCs) are typically quiescent but undergo cell division following large-scale OSN loss to replace OSNs and sustentacular cells. Markers are based on [5,6,10,20,21,22,23,24,25]. (**C**) Neural stem cells in the SVZ generate neuroblasts, which migrate via the rostral migratory stream (RMS) to reach the olfactory bulb (OB), where they migrate radially and differentiate into multiple molecularly defined subtypes of granule cells (GCs) and inhibitory juxtaglomerular neurons (JGNs). Inset: different regions of the SVZ generate distinct subclasses of postnatally born OB neurons [26]. (**D**) Schematic of cell types involved in postnatal SVZ neurogenesis. Created with BioRender. Modified from Figure 1 of [27].

**Figure 2:**
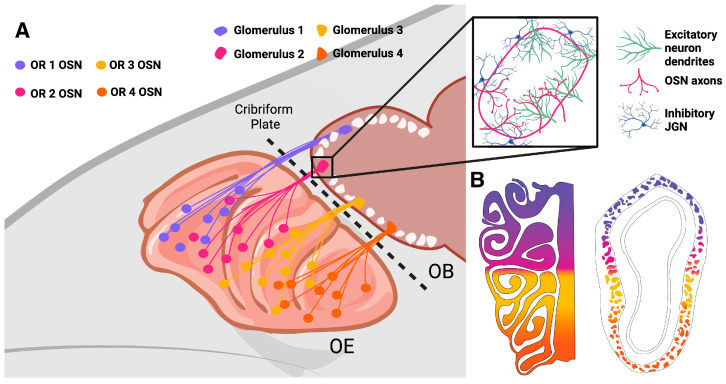
Schematic of the nose to brain pathway for odor information in rodents. (**A**) OSNs located in the OE at the back of the nose project through the cribriform plate to the ipsilateral OB. The axons of OSNs expressing the same odorant receptor (OR) coalesce together to form glomeruli, thereby generating a highly organized odor input map to the OB. Note that only a single glomerulus per OR is shown here, whereas OSNs expressing each OR form on average two glomeruli per OB in mice. The inset on the right shows an expanded view of a glomerulus, containing OSN axons and dendrites of mitral/tufted cells and inhibitory JGNs. Note that glomeruli are compartmentalized into axodendritic and dendrodendritic domains [28]. Created with BioRender. (**B**) Schematic showing coronal view of the left side of the OE and left OB. The shading illustrates the classical zonal organization of the OE: OSNs expressing each OR are typically scattered within a single zone or longitudinal band of the OE [29,30,31]. Note, however, that not all ORs are restricted to a single zone; rather, expression domains for each OR are continuous and overlapping [32,33,34]. Nevertheless, projections from the OE to the OB are topographic, with the position of glomeruli along the dorsal to ventral axis of the OB corresponding to the location of OSNs along the dorsomedial to ventrolateral axis of the OE [33,35].

**Figure 3:**
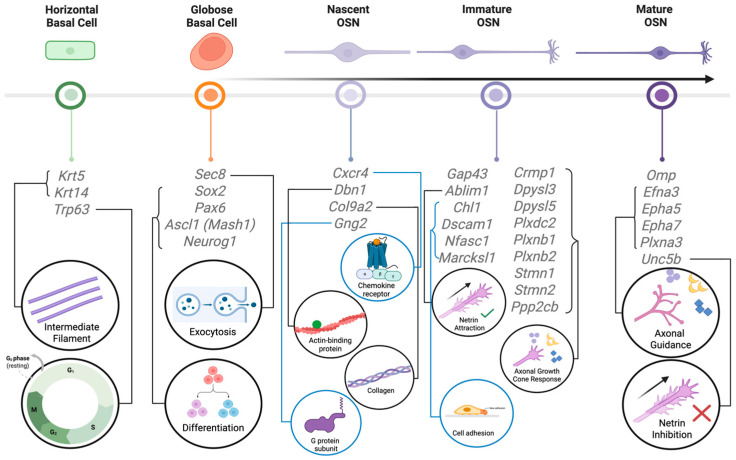
Cell types in the OSN lineage. The schematic shows markers expressed by OE basal cells and key OSN maturational stages, as well as the major functions of those genes. Markers are based on data from [7,9]. Created with BioRender.

A unique feature of the rodent olfactory system is that postnatally born neurons, generated from neural stem cells in two distinct locations, continue to integrate into the highly ordered circuits of the olfactory bulb (OB) throughout life (Figure 1). Olfactory sensory neurons (OSNs), which detect odors in inhaled air and transmit this odor information to the OB, have long been known to be generated throughout life. The first evidence for the generation of new OSNs in adults, in this case following axotomy, appeared as early as in 1940 [1], and this was confirmed in mice and rats several decades later [2,3]. In the healthy olfactory epithelium (OE), globose basal cells (GBCs) undergo mitosis to generate Ascl1-expressing neuronally committed intermediate cells [4]. These cells rapidly become nascent OSNs that express CXCR4 and DBN1 before transitioning to immature OSNs that express growth-associated protein 43 (GAP43) and G-protein γ-subunit (Gγ8) [5,6,7,8,9] (Figure 2 and Figure 3). The onset of OSN maturity is defined by the expression of the olfactory marker protein (OMP) and concomitant downregulation of the expression of GAP43 and Gγ8 [10,11,12]. The entire maturation process of an OSN is completed within 7–8 days after cell division [13,14,15]. GBCs generate OSNs in both the healthy OE and following olfactory nerve lesion [16]. The role of horizontal basal cells (HBCs), which are normally quiescent [17], in postnatal OSN neurogenesis has been debated, with one study finding HBC-derived OSNs only after extensive OE lesion [18] and another showing that HBCs contribute to OSN neurogenesis both under normal conditions and following OE or OB damage [19]. This discrepancy likely arises from differences in labeling density between the two studies, and because HBC-derived neurogenesis was concentrated in temporal waves, one shortly after birth and another peaking at 4 months of age [19], making the age of experimental mice a key factor for detection.

Neurogenesis in the mammalian central nervous system was long thought to be restricted to embryonic development [36]. However, evidence for adult neurogenesis in the rodent brain began to emerge in the 1960s, with the discovery of newly generated granule cells in the dentate gyrus of the postnatal rat hippocampus [37] and the OB [38]. It is now well established that a high level of neurogenesis persists postnatally in the rodent subventricular zone [39,40], with distinct sub-regions of the subventricular zone (SVZ) giving rise to molecularly distinct subtypes of OB interneurons [26] (Figure 1C,D). While the rate of neurogenesis does decline with age [41,42], most OB neurons are generated postnatally [43,44]. Astrocytes residing in the SVZ act as neural stem cells that then give rise to transit-amplifying cells, which differentiate into neuroblasts [45,46]. Neuroblasts generated in the SVZ, which lines much of the walls of the lateral ventricle, undergo chain migration via the rostral migratory stream (RMS) to the OB [38,47,48,49,50,51,52]. There, they migrate radially from the subependymal layer of the OB (also called the OB core or RMS-OB) as they differentiate into multiple subtypes of granule cells (GCs) and inhibitory juxtaglomerular neurons (JGNs) in the glomerular layer (GL). Tens of thousands of neuroblasts arrive in the OB each day, with the vast majority becoming GCs but a small subset differentiating into GL inhibitory neurons [53,54,55,56,57]. There is also evidence that a much lower level of neurogenesis occurs in the RMS and the core of the OB itself to generate calretinin (CR)- and tyrosine hydroxylase (TH)-expressing OB neurons [26,58,59,60,61,62,63,64,65]. The contribution of local neurogenesis decreases markedly after the first postnatal week, such that SVZ neurogenesis makes a much greater relative contribution to OB neurogenesis in adult vs. early postnatal mice [64]. However, OB core neurogenesis was strongly upregulated following OSN axotomy in adult mice [66], suggesting that it can still contribute under specific circumstances.

Here, we focus on the roles of postnatally generated neurons in the rodent olfactory system. Given that both the generation of new neurons and their integration and function in the nervous system are energetically expensive [67,68], what are the benefits of maintaining postnatal neurogenesis? We examine evidence for whether postnatally born neurons in the olfactory system add functions or enhance plasticity in the healthy brain, as well as their roles in neuronal replacement and regeneration of damaged olfactory circuits. We address these questions for OSNs, JGNs and GCs, the three major classes of postnatally generated neurons in the rodent olfactory system.

## 2. Postnatal Neurogenesis of OSNs

### 2.1. Dynamics of OSN Neurogenesis

OSN neurogenesis persists throughout life in all terrestrial mammals. OSNs have a vulnerable location in the OE, where they can be directly exposed to physical and chemical damage as well as viruses. The half-life of OSNs is about a month, with few surviving beyond three months except under specific environmental conditions that prevent damage [69,70,71]. Therefore, continuous replacement is necessary to maintain olfactory function. The rate of OSN proliferation is highest in young postnatal mice, with generation of immature OSNs peaking in two-week-old mice [11,72]. OSN neurogenesis then declines substantially by one month of age, with further reductions in older adults, nevertheless persisting at a low level even in 24-month-old mice and 32-month-old rats [11,73,74]. Despite this constitutive neurogenesis, relatively few OSNs survive and functionally integrate into OB circuits [11,13,75,76,77]. Furthermore, OSN cell death is elevated in immature and young mature (less than 14-day-old) OSNs.

### 2.2. Activity Dependence of OSN Neurogenesis

There is substantial evidence that OSN survival is activity dependent. OSNs that were naturally less active had shorter lifespans than those that were more active [78]. Furthermore, OSNs that lacked OCNC1, rendering them unable to transduce odor binding, became depleted from the OE [79]. Odor stimulation has been shown to promote survival of OSNs expressing the cognate odorant receptor (OR) by preventing apoptosis [80,81,82] and even to increase generation of OSNs expressing the cognate OR [83,84]. This latter finding is surprising given the stochastic nature of OR selection by OSNs [85]. 

However, cognate odor exposure has also been shown to decrease the density of OSNs expressing some ORs but not others [86,87]. The effects of naris occlusion, which decreases sensory input to OSNs, also appear to depend on the OR that is expressed as well as the age of the mouse. Neonatal unilateral naris occlusion reduced the number of OSNs in the ipsilateral OE of three-week-old mice in one study [87], while another study found that OSN density could increase, remain unchanged or decrease, dependent on the OR expressed [88]. Indeed, generation of OSNs expressing ORs that detect male musk odor was strongly and selectively reduced by naris occlusion, with generation of OSNs expressing other randomly selected ORs being unaffected [84]. In contrast, three weeks of naris occlusion in adult mice did not affect OSN density, proliferation or apoptosis rates [89]. Similarly, while there was no overall change in OSN apoptosis, two-week-old mice that underwent two weeks of unilateral naris occlusion exhibited selective increases or decreases in the generation of OSNs that expressed specific ORs [83]. There were also age-dependent differences in whether the abundance of OSNs expressing specific ORs was affected by naris occlusion. Therefore, it is apparent that while activity can regulate OSN density, the effect of activity manipulations is OR-specific and varies across postnatal development. Hence, it appears that the repertoire of OSNs can be tuned to reflect recent sensory experience.

OSNs project their axons through the cribriform plate to provide odor input to neurons in the OB. While OSNs expressing a particular OR are scattered throughout a large domain of the OE, the axons of OSNs expressing the same OR coalesce to form two glomeruli per OB, generating an ordered odor input map to the brain (Figure 2). There is evidence that OSN survival is modulated by the OB; indeed, synapse formation with OB neurons has been proposed to be essential for OSN survival [90]. Supporting this hypothesis, olfactory bulbectomy, which removes the normal postsynaptic targets of OSNs, resulted in massive cell death of newly generated OSNs before they reached two weeks of age [75]. Furthermore, OB principal neuron activity also regulated OSN survival in an activity- and OR-specific manner, with increased mitral cell (MC) excitability rescuing OSNs expressing the M72 OR from cell death due to naris occlusion or odor enrichment [87]. However, the mechanism by which MC activity can regulate OSN survival remains unclear.

### 2.3. Roles for Immature OSNs

To fully understand how ongoing OSN neurogenesis impacts olfaction, it is important to determine when newborn OSNs become functionally relevant. The prevailing view had been that OSNs must be mature, i.e., express OMP, to contribute olfactory information to OB circuits. However, an accumulating collection of evidence suggests that in fact, newly generated OSNs are functional while still immature.

OSNs begin to express ORs four days after terminal cell division, which is several days before GAP43 expression is downregulated and OMP expression begins [13,15,91]. A subset of “late immature” OSNs also express all the signal transduction machinery necessary to generate action potentials in response to odor binding [92]. Immature OSN dendrites lack the sustentacular cell wrapping that occurs in their mature counterparts but nevertheless achieve sufficient interaction with sustentacular cells via strong expression of focal adhesion proteins [93,94,95,96]. However, immature OSN dendrites have short cilia, with OSN maturation necessary for the full extension of cilia that is thought to be important for odor detection [75,97]. Whether immature OSNs can detect odors was recently tested directly using in vivo 2-photon calcium imaging of odor-evoked responses, finding that immature Gɣ8+ OSNs could detect and transduce odorant binding [98]. However, single cell RNA sequencing studies found that some immature OSNs express mRNA transcripts encoding more than one OR [92,99]. ORs not only determine the odors that an OSN can detect but also play an important role in OSN axon guidance. Therefore, odor input from OSNs expressing multiple ORs could result in glomeruli being more broadly tuned to odors and so could potentially degrade the highly organized glomerular map. Importantly, 2-photon calcium imaging showed that odor selectivity based on a panel of 15 odors was similar in immature and mature OSNs [98], possibly because additional regulatory mechanisms ensure that only a single OR protein is translated, or because expression of low levels of additional ORs has little functional impact.

Interestingly, immature OSNs provided information about odor concentration that was distinct from, but complementary to, that provided by mature OSNs. Specifically, immature OSNs encoded information about differences high in the concentration range, which is not available from mature OSNs because their responses have already saturated [98]. There is also strong evidence that immature OSNs can transmit odor information to OB neurons. Synapse formation by immature OSNs is exuberant [100]. Furthermore, the synapses formed by immature OSNs are morphologically mature, and selective optogenetic stimulation of immature OSNs results in robust firing of excitatory OB neurons in vivo and elicits monosynaptic responses in superficial tufted cells (TCs) ex vivo [89,98]. Finally, immature OSNs enabled mice to perform odor detection and simple odor discrimination tasks in the absence of mature OSNs, supporting the conclusion that they provide behaviorally relevant odor information to the OB [98]. It will be important to determine the specific contribution of immature OSNs during development, in healthy adult circuits and during regeneration. Indeed, the finding that turnover of OSN presynaptic terminals was three-fold higher for immature vs. mature OSNs [89] may suggest that integration of newborn OSNs creates a window of heightened plasticity for adaptation of OB circuits to current environmental conditions.

### 2.4. OSN Neurogenesis Following OE Damage

**Figure 4:**
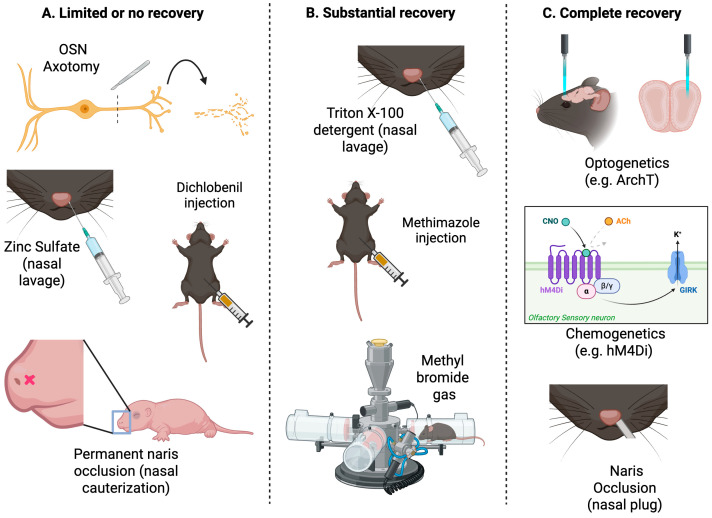
Different experimental manipulations to reduce OSN sensory input to the OB result in differing degrees of recovery. (**A**) Techniques that cause irreversible damage to the OE (OSN axotomy, zinc sulfate and dichlobenil) or permanently block OSN sensory input to the OB (nasal cauterization). Recovery of OSN projections to the OB is limited or absent, resulting in significant olfactory deficits. See text for applications of these techniques. (**B**) Techniques that ablate OSNs but do not damage OE stem cells, enabling substantial OSN repopulation and regeneration of OSN projections to the OB. However, axonal targeting may be error prone. (**C**) OSNs immediately recover from optogenetic or chemogenetic silencing. Nasal plug removal restores sensory input to the OB after a period of naris occlusion. Chemogenetics schematic BioRender. Adapted from [106]. Figure created with BioRender.

The capacity of OSN neurogenesis to restore the OE after damage has been extensively studied, with a smaller number of studies investigating the extent to which sensory input to the OB and readouts of olfactory function are restored. A range of methods have been used to assess the capacity for OSN regeneration, some of which result in irreversible loss of all OSNs, due to loss of basal cells (Figure 4). Examples of this are zinc sulfate treatment, which causes complete loss of all cell types in the OE and therefore results in persistent deficits in OSN density and therefore OB reinnervation that appear irreversible, being present several months later [101]. Treatment with the pesticide dichlobenil also damages GBCs and HBCs, which limits the extent of OE regeneration and complicates interpretation of OB reinnervation experiments [102,103,104]. Dichlobenil treatment has dose-dependent effects on OE regeneration, with a low dose selectively and permanently ablating OSNs in zone 1 (Figure 2B) of the OE, while higher doses also damage other OE zones but permit some regeneration [102,103,105].

Here, we focus on methods that do not damage basal cells and hence permit regeneration to occur. The effects of treating the OE with the detergent Triton X-100 are reversible, with OSN repopulation occurring within 6 weeks [107]. Similarly, following ablation of virtually the entire OSN population using either inhalation of methyl bromide (MeBr) gas or systemic administration of methimazole (MMZ), basal cell division can completely repopulate much of the OE with OSNs expressing a normal OR repertoire even in adult mice [32,108,109]. Importantly, treatment with either MeBr or MMZ downregulates p63 expression in HBCs, which is both necessary and sufficient for their activation [22,110]. These activated HBCs can generate all OE cell types, including persistently active GBCs [18,110,111,112,113]. Cell fate is regulated by the canonical Notch signaling pathway, with only OSNs being generated in the absence of redundant NOTCH1 and NOTCH2 receptor signaling [114]. SOX2 signaling in activated HBCs is also important for generation of OSNs rather than sustentacular cells [113].

MeBr treatment has dose-dependent effects on the OE [115,116], primarily affecting sustentacular cells and mature OSNs while basal cells are generally preserved [109,116]. Regeneration of the OE begins as early as three days after initial exposure, and even during ongoing exposure, and the OE is repopulated with OSNs ten weeks post-exposure, albeit with some persistent damage after high doses [116,117,118]. MMZ, an anti-thyroid drug, selectively ablates OSNs without damaging progenitor cells in the OE [119,120,121], effectively removing the ability to detect odors [98,122] without impairing the capacity for OSN neurogenesis. Mature OSNs are present as early as seven days after MMZ treatment and reach control levels around 4–12 weeks post-treatment, with higher doses requiring longer recovery times [98,108,123,124,125].

Basal stem cells therefore enable a normal complement of OSNs to be regenerated. However, to restore olfactory function, they need to accurately reinnervate the OB. Indeed, there is evidence for substantial anatomical regeneration of the OB glomerular map following either MeBr or MMZ treatment [118,122,126]. However, re-innervation of the OB after OSN ablation is not error free: OSNs expressing some ORs projected to inappropriate and/or a larger than normal number of locations in the glomerular layer [103,122,126,127,128]. Furthermore, a recent study found a specific deficit in OSN reinnervation of dorsomedial glomeruli, even 20 weeks post-MMZ [129]. These inaccuracies likely arise because a specialized population of navigator OSNs, which ensures accurate OSN axon targeting to glomeruli during development, is present only during embryonic and early postnatal life [130].

Given this incomplete anatomical recovery, it is not surprising that there is mixed evidence for functional recovery following MeBr or MMZ treatment. 12 weeks after MeBr-mediated OSN ablation, mature OSN presynaptic terminals recovered near-normal levels of odor responsiveness, with odorants evoking responses in glomerular foci within the same functional domains of the GL as before ablation [127]. However, these regenerated foci, while exhibiting odor-specific responses, were small and lacked clear glomerular boundaries. Studies using MMZ-mediated ablation provide mixed evidence for functional recovery. Functional reconnection of newly generated OSN axons with OB neurons occurs within 16 days of MMZ treatment [124]. These new synapses had high presynaptic release probability as soon as they formed, although maturation during the ensuing week strengthened presynaptic release even further. Functional restoration of a previously learned odor discrimination task was also seen 45 days after MMZ-mediated OSN ablation [122]. In contrast, a recent study reported poor recovery of odor-evoked responses on the dorsal surface of the OB, with deficits being most pronounced in the dorsomedial region, weeks to months after MMZ treatment [129]. The regional nature of this deficit provides a likely explanation for the discrepancy with previous studies, which either may not have sampled from the dorsomedial region or involved glomeruli that did regenerate successfully.

## 3. Postnatal Neurogenesis of OB GL Neurons

### 3.1. Integration of Adult-Born OB GL Neurons

**Figure 5:**
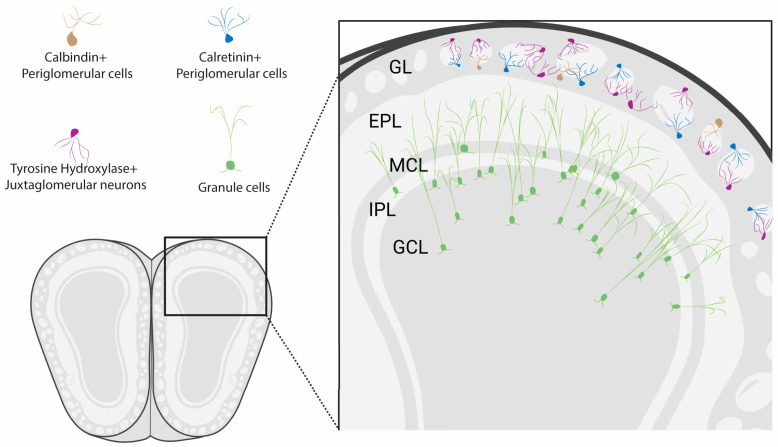
Postnatally generated inhibitory neurons in the OB. The major postnatally born inhibitory JGN subtypes can be distinguished by their non-overlapping expression of the markers calretinin, calbindin and tyrosine hydroxylase. Postnatally born GCs are found in the mitral cell layer (MCL), internal plexiform layer (IPL) and granule cell layer (GCL). While there are some studies of GC subtypes that express specific molecular markers, there is considerable overlap between some markers, and most studies have focused on the postnatally generated GC population as a whole. Partially created with BioRender.

Postnatally born JGNs exhibit voltage dependent conductances and synaptically integrate into circuits in the OB shortly after they arrive at their final destination in the GL [56,131] (Figure 5). Spontaneous Ca^2+^ transients are ubiquitous in newborn JGNs as soon as they arrive in the GL, with spontaneous activity peaking when neurons are 3–4 weeks old [132]. Functional integration is remarkably rapid: over half of adult-born JGNs respond to odor stimulation within two days of arriving in the GL, even while they are still migrating locally within the GL [133]. Hence, adult-born JGNs can very rapidly begin to contribute to olfactory processing. However, there is conflicting evidence as to whether mature adult-born neurons are functionally distinct. One study found that odorant response amplitudes and sensitivity are similar to those of surrounding resident JGNs [133], whereas another reported differences in odor-evoked response amplitude and reliability between adult-born and resident JGNs, the direction of which differed between awake and anesthetized mice [134].

Postnatally generated JGNs undergo significant morphological and functional changes as they mature. Soon after they arrive in the GL, aspiny adult-born JGNs undergo large-scale dendritic reorganization as they mature to generate more elaborate dendritic trees, while spiny dendrites are stable but exhibit a high level of dendritic spine dynamics [135]. The initial increase in adult-born JGN dendritic complexity is accelerated by odor exposure [136]. Later in maturation, there is a subsequent reduction in dendritic complexity [137]. Interestingly, mature adult-born JGNs retain structural plasticity of distal dendrites [135] and continue to form and eliminate synapses (based on PSD puncta dynamics) [136], albeit at a lower level when they are immature. Furthermore, long-term tracking of adult-born JGNs over several months showed that their morphology alters to produce more compact dendritic tree, but that this is a consequence of mouse (rather than neuronal) age [138]. Importantly though, an increase in the density of PSD95 puncta maintained the total number of synapses in these older JGNs.

Functionally, there is also significant postsynaptic maturation at OSN inputs to adult-born JGNs. There was a significant increase in AMPA:NMDA ratio and a reduction in the contribution of NR2B-containing NMDA receptors with maturation [139]. Newly arrived adult-born JGNs may therefore respond differently to OSN input compared to fully mature JGNs, hence their circuit function may transiently be distinct. This idea is corroborated by the finding that odor responsiveness of adult-born JGNs peaked during their development before reducing at maturity [140]. Interestingly, odor exposure during JGN maturation enhanced odor selectivity once JGNs in the OB region activated by these odors were mature, suggesting that the processing of odor information by mature adult-born JGNs is based on past experience.

There is conflicting evidence as to whether postnatal neurogenesis results in JGN turnover or adds to the population, with reports of both balanced addition and elimination [141], and constant net addition throughout at least nine months [142]. Nevertheless, survival of adult-born JGNs is dependent on circuit integration [143] and is regulated by activity: it is promoted by sustained odor enrichment [144], whereas naris occlusion reduces the survival of adult-born JGNs generated either before or during loss of input [145]. Interestingly, low spontaneous activity also correlated with increased survival of adult-born JGNs, whereas the morphologies, and amplitudes and time courses of odor-evoked responses, were similar in adult-born JGNs that survived vs. died [137].

Overall, there are some discrepancies between studies that considered postnatally born JGNs as a single population, which may partly arise from differences in labeling strategies, time courses and experimental approaches. However, differences between subtypes of postnatally generated JGNs are also likely to be a key factor. In the following sections, we focus on studies assessing the three major subtypes of JGNs that undergo postnatal neurogenesis, as defined by the non-overlapping expression of the molecular markers CR, calbindin (CB) and TH.

### 3.2. Calretinin- and Calbindin-Expressing Periglomerular Cells

CR+ and CB+ periglomerular cells (PGCs) undergo postnatal neurogenesis, with CR+ PGCs being generated in the anterior dorsal and septal regions of the SVZ and CB+ PGCs mostly being generated in the ventral SVZ [26,146]. Interestingly, CR+ PGC neurogenesis peaks around the time of birth whereas CB+ neurogenesis peaks during embryogenesis [147,148,149,150]. There is some discrepancy amongst studies regarding the extent of adult CB+ PGC neurogenesis, but overall, there appears to be a low level of ongoing CB+ PGC neurogenesis up to postnatal day (P) 60, but not later [142,147,149,150,151]. In contrast, CR+ PGCs show a much higher level of continued neurogenesis, persisting until mice are at least two years old [42,142,149,150,151].

#### 3.2.1. Properties of Calretinin-Expressing PGCs

CR+ PGCs are the most numerous GL neuron type and have typical PGC morphology: they are anaxonic with a polarized dendritic tree ramified in a single glomerulus [146,152,153]. However, their synaptic and electrophysiological properties are unique [146]. CR+ PGCs do not receive synaptic inputs from OSNs [154,155], but do receive centrifugal cholinergic and inhibitory inputs [156,157] and excitatory input from MCs and TCs [146]. Surprisingly, these excitatory synapses are functionally immature, with a high density of NMDA receptors, and hence far lower AMPA:NMDA receptor ratios than CB+ PGCs [146]. CR+ PGCs also provide inhibitory input onto MCs and TCs [158].

Integration of CR+ neurons into GL circuits does not progress over time, leading to the proposal that they are a pool of latent neurons that could be recruited into OB circuitry as needed [146]. It has also been suggested that CR+ PGCs may be able to convert into another PGC subtype [146], although there is no direct evidence for this. Depolarizing current injections in CR+ PGCs induce at most a single action potential (AP) before the neurons enter an inactivated state with only passive depolarization occurring for the rest of the current step [146,159]. As a result, CR+ PGCs cannot respond to repetitive or structured stimuli, limiting their inhibitory influence on MCs and TCs. However, their high input resistance and very high capacitance (over 4 pF) implies that they may be activated by weak excitatory input, including noise transmitted disynaptically from the olfactory nerve [159]. Hence, a role in improving signal: noise ratio in OB circuitry has been suggested for CR+ PGCs [159]. Notably, these studies were conducted in CR-EGFP lines, so the recorded neurons represent a mixed population of embryonically and postnatally generated CR+ PGCs. Therefore, while there are unlikely to be large differences, it remains to be determined experimentally whether neuronal birth date impacts CR+ PGC functional properties.

#### 3.2.2. Properties of Calbindin-Expressing PGCs

CB+ PGCs extend one or more dendritic process into a glomerulus and thin processes from the soma into periglomerular or intraglomerular regions [160]. CB+ PGCs rarely receive OSN synaptic input [153,155], but, like CR+ PGCs, receive centrifugal cholinergic [156] and inhibitory inputs [157]. Also similar to CR+ PGCs, they provide inhibitory input to MCs and TCs [158,161]. In Kv3.1-EYFP mice, in which EYFP is expressed in CB+ PGCs and other minor GL subtypes but not CR+ PGCs or TH+ GL neurons, Kv3.1-EYFP+ PGCs responded to OSN stimulation with a short barrage of summating EPSCs that lasted tens of milliseconds and to stimulation of single principal neurons projecting into the same glomerulus [154]. CB+ PGCs may therefore contribute to intraglomerular lateral inhibition, which promotes spike timing variability amongst principal neurons projecting to the same glomerulus. Again, it remains unclear whether there are functional distinctions between embryonically and postnatally generated CB+ PGCs.

#### 3.2.3. Role of Postnatal Neurogenesis of Calretinin- and Calbindin-Expressing PGCs

Several studies have identified a range of molecular mechanisms by which postnatal addition of CR+ cells and/or CB+ cells can be disrupted [162,163,164,165,166,167,168,169]. However, a challenge in interpreting these studies is that the manipulations were constitutive, making it unclear whether observed changes in CR+ and/or CB+ PGCs were due to altered embryonic, rather than postnatal, neurogenesis. Furthermore, none of these studies investigated the impact of altered CR+ and/or CB+ PGC density on any aspect of olfactory function.

Several studies have investigated the effects of loss of sensory input on postnatal generation and survival of CR+ and CB+ neurons. An early study in rats showed that CB+ neurons but not CR+ neurons are sensitive to loss of odor input due to naris occlusion [170]. Naris occlusion did not affect the density or soma area of CR+ PGCs, whereas CB+ PGCs showed reduced staining intensity, density, and soma size. More recent studies have not found similar changes in CB+ PGCs in mice. One study found that neither odor enrichment nor naris occlusion selectively modulated the survival of CR+ or CB+ PGCs [144]. In agreement with this, another study found that odor-evoked activity is not essential for CR+ or CB+ PGC fate determination or maintenance in mice [145]: naris occlusion did not alter the percentage of BrdU-labeled cells that expressed CB or CR in the GL, whether BrdU was administered before (fate maintenance) or after (fate determination) naris occlusion. Subsequently, another study found no effect of prolonged odor enrichment on the percentage of newborn GL neurons that expressed CR or CB [171]. Collectively, these three studies suggest that neurogenesis and survival of CR+ and CB+ PGCs are unaffected by manipulations of olfactory input. A recent study, which used methimazole treatment to ablate OSNs found a transient increase in CR+ PGC density 7 days post-treatment [125]. Follow-up experiments suggested that this rapid increase in CR+ neuron density was due to increased CR protein expression levels [125], adding further support to the idea that CR+ PGC neurogenesis is not affected by altered odor input.

Overall, how postnatal neurogenesis of CB+ and CR+ PGCs impacts olfactory processing in the healthy adult brain remains unclear. The impact of addition of small numbers of CB+ PGCs up to two months of postnatal age remains to be determined, but they may strengthen glomerular layer lateral inhibition. In contrast, CR neurogenesis occurs at a much higher level, but the resultant neurons have little impact on OB processing. It remains an open question as to whether or how postnatal neurogenesis of CR+ and CB+ PGCs adds new functions. In the context of regeneration, the transient increase in CR+ neuron density seen after MMZ treatment supports the intriguing possibility that CR+ PGCs may play a greater role in the regenerating OB than in the healthy OB, as suggested previously [146]. Electrophysiological recordings of CR+ PGC neuron excitability, inputs and outputs shortly after MMZ treatment could test this possibility. Furthermore, additional data are needed to determine what role, if any, is played by CB+ neurogenesis during regeneration of sensory input to the OB.

### 3.3. Postnatal Neurogenesis of Dopaminergic Neurons

Due to the absence of neurons that express other catecholamines in the OB, expression of TH, the rate limiting enzyme in dopamine biosynthesis, enables identification of dopaminergic (DA) neurons in the OB. These DA neurons are located predominantly in the GL with a few scattered in the external plexiform layer(EPL) [172]. Over 97% of these TH+ neurons co-express GAD67 [173]. Although DA neurogenesis peaks embryonically, DA neurons continue to be generated postnatally in the dorsal region of the SVZ for at least two years [26,42,147,148,149,150,151,174].

#### 3.3.1. Structure and Function of DA Neurons

Classification of DA neurons has been controversial [175,176,177,178]. OB DA neurons are not a homogeneous population: they differ in soma size, dendritic and axonal morphology, location within the GL, and whether they receive OSN input [172,173,176,179,180,181]. It is well established that there are two sub-classes of OB DA neurons that can be defined by their soma size [155,181,182]. Interestingly, large DA neurons, which have an axon, are generated only embryonically, whereas small DA neurons, which are anaxonic, continue to be generated postnatally [176,183]. DA neurons can project hundreds of microns within the GL and can contact many glomeruli [173,184]. They have been divided into two morphological types based on the number of glomeruli that they contact: oligoglomerular, contacting < 18 glomeruli; and polyglomerular, contacting at least 30 glomeruli [173]. DA neurons have also been classified into five groups based on their structure [180], but understanding what this means for their function will require mapping of synaptic inputs and outputs for each group.

Different subtypes of DA neurons can also be defined by their synaptic inputs: 30% receive monosynaptic OSN input, while the other 70% are instead driven by disynaptic excitation via external tufted cells (ETCs) [173]. Interestingly, all OSN-driven DA neurons are oligoglomerular, whereas ETC-driven DA neurons contact more glomeruli and can be oligoglomerular or polyglomerular [173]. Another key feature of DA neurons is that up to 80% of them exhibit a high level of spontaneous firing, which is present both in acute OB slices and after dissociation, indicating that this is an intrinsic property [181,185,186]. In OB slices, spontaneous firing occurs at ~8 Hz and requires both a persistent sodium current and a T-type calcium current, resulting in pacemaker activity [181,186].

DA neurons co-release dopamine and GABA from separate pools of synaptic vesicles [187,188,189], suggesting that they have dual signaling roles. Surprisingly, it has not been directly determined whether this is true of both large and small DA neurons. However, indirect evidence suggests that co-release does occur in both subtypes. First, given the prevalence of co-release and that 85% of DA neurons are of the small subtype [181,190], it is highly unlikely that none of them exhibit co-release. Second, co-release by DA neurons onto ETCs in other glomeruli [189] suggests that large axon-bearing DA neurons, which project their axons over long distances within the GL [184], are capable of co-release. Hence, differences in the function of embryonically vs. postnatally generated DA neurons are likely to arise primarily from differences in their morphology, which determines intrinsic properties as well as input and output connectivity.

Given their morphological and physiological diversity, it is not surprising that DA neurons have been implicated in numerous functions in odor processing. These include reducing OSN presynaptic release probability [191,192,193,194,195], inhibition of OB output [196], gain control and decorrelation of odor representations in MCs and TCs [197], inhibition of PGCs and biphasic inhibitory-excitatory modulation of ETCs in distant glomeruli [189,198]. It remains unclear which of these functions are specific to embryonically vs. postnatally generated DA neurons. Interestingly, though, postnatally generated DA neurons exhibit stronger and more finely tuned odorant responses than their embryonically generated counterparts [176], suggesting that they may indeed play different functional roles in odor processing.

#### 3.3.2. Activity Dependence of DA Neuron Survival and Function

Initial studies found that both TH expression and dopamine production are strongly down-regulated when sensory input is lost due to chemical lesion of the OE, surgical deafferentation of the OB or naris occlusion [199,200,201,202,203]. At least for naris occlusion, downregulation of TH expression and DA production occurs in both newly generated and resident DA neurons [144], and occurs within one day [204]. In contrast, dopamine decarboxylase (DDC) expression and activity were unaffected by loss of sensory input [201], which, coupled with the recovery of TH expression several weeks after reversible chemical lesion of the OE, led to the suggestion that DA neurons survive loss of sensory input [199,200,201]. However, these studies were performed before the discovery of adult OB DA neurogenesis [26,147,148,149,150,151,174]. More recent studies employing Cre-mediated recombination to indelibly mark DA neurons with fluorescent proteins showed that DA neurons are indeed dependent on sensory input for survival [205,206,207]. The first study to use this approach employed a histological approach in TH-Cre mice, finding that a vulnerable subpopulation (~40%) of DA neurons die in the absence of sensory input, whereas the remaining ~60% are resilient through four weeks’ naris occlusion [206]. A subsequent study, again using a histological approach but using different TH-Cre and reporter lines, found a similar magnitude of DA neuron loss due to naris occlusion [205]. In contrast, a recent study that employed in vivo 2-photon imaging to track individual DA neurons before and after four weeks of naris occlusion found only ~6% loss [207]. A key difference in this study is that a cohort of perinatally born neuroblasts was labeled via dorsal SVZ electroporation, with DA neurons being identified by TH-GFP co-expression prior to naris occlusion. Hence, it is possible that either birth date or susceptibility to electroporation selects for a subpopulation of more stable DA neurons. Alternatively, or in addition, histological approaches may overestimate the magnitude of DA neuron loss due to inter-animal variability. Returning to the idea that maintained DDC expression argues against DA neuron loss, there is recent evidence that DDC expression may be up-regulated following an early, transient reduction, suggesting that increased DDC expression in remaining DA neurons at later time points after sensory input loss could compensate for the loss of others. Further studies are therefore needed to resolve the extent of DA neuron loss in the absence of sensory input.

#### 3.3.3. Activity Dependence of DA Neurogenesis

Prolonged odor enrichment for 9 weeks expanded the GL DA neuron population by ~20%: the number of newborn DA neurons increased significantly, resulting in newborn neurons accounting for a larger proportion of the total DA neuron population [171]. This therefore suggests that odor enrichment drives increased integration of adult-born DA neurons. Another study found that very few TH+ GL neurons, born before or after onset of naris occlusion, were present. This was interpreted to suggest that sensory input is important not only for establishment of DA cell fate, but also for maintenance of this phenotype. However, the well-established activity dependence of TH expression, coupled with evidence from other studies that surviving DA neurons can re-express TH [207] suggests that TH expression cannot provide a reliable readout of DA neuron generation or survival.

Interestingly, both histological and in vivo chronic imaging approaches have found that restoration of sensory input after naris occlusion enabled DA neurons to repopulate [206,207]. However, it remains unknown whether DA neurogenesis is upregulated following naris reopening, or whether constitutive levels of DA neurogenesis are sufficient to enable repopulation within four weeks.

#### 3.3.4. Role of Postnatal Neurogenesis of OB DA Neurons

An interesting feature of adult DA neurogenesis is that it occurs against a backdrop of very low levels of DA neuron cell death [174] and hence does not serve only to replace lost neurons. What functions are being added or improved by the continual addition of GL DA neurons? A conclusive answer to this question would require a detailed understanding of the specific functions of the small, anaxonic DA neurons that continue to be added postnatally. Nevertheless, the absence of an axon limits the influence of postnatally generated DA neurons to intraglomerular, rather than interglomerular, inhibition. This can, however, impact both OSN presynaptic terminals and projection neuron dendrites, and hence modulate both OB input and output. Therefore, newborn DA neuron integration may enable continuous re-optimization of OB circuitry to current environmental conditions. Another open question is whether newly generated DA neurons exhibit heightened plasticity as they integrate, as has been found for OB GCs [208]. Hence, further studies are needed to understand how continued DA neurogenesis enhances olfactory processing.

## 4. Granule Cells

### 4.1. Postnatal Generation and Maturation of GCs

GCs are the most abundant neuronal type in the OB and are found in the granule cell layer (GCL), mitral cell layer (MCL) and internal plexiform layer (IPL) [209,210,211] (Figure 5). They are small anaxonic cells, and their dendrites bear large spines, or gemmules, where they form reciprocal dendrodendritic synapses with the lateral dendrites of MCs and TCs [212]. They also comprise the vast majority of adult-born neurons in the OB [53]. GCs are generated throughout the SVZ in both early postnatal and adult mice [26]. Most deep GCs are generated from ventral regions, whereas superficial GCs tend to be generated from dorsal regions. Interestingly, CR+ GCs are generated in the same anterior regions that produce CR+ PGCs. While GC neurogenesis persists throughout life in mice, the rate of neurogenesis decreases substantially with aging [42,213,214,215,216]. Indeed, the number of migrating neuroblasts decreases by over 40% between two and four months of age, with further reductions through the first year of life [41]. Surprisingly, this reduction occurs downstream of neural stem cell proliferation in the SVZ, which was maintained in two-year-old mice despite a reduction in the overall number of neural stem cells [217].

Postnatally born GCs exhibit voltage dependent conductances soon after they reach the OB [56,131], and contact all major types of OB neurons [218]. The timeline for structural and functional maturation of newborn GCs has been well characterized. Newborn GCs pass through five morphological stages, exhibiting dendritic spines as early as 14 days after birth, and showing mature morphology, such as elaborate dendritic arbors, by 30 days [219]. Adult-born GCs express extrasynaptic GABA_A_ and AMPA receptors while still migrating through the RMS, and NMDA receptor expression begins upon arrival in the GCL [131]. Indeed, GABAergic signaling plays an important role in adult-born GC structural maturation [220]. Functional maturity accompanies morphological maturity: functional output synapses are present as early as two weeks after birth [218], and synapses on dendritic spines are functional by four weeks after birth [131]. These adult-born GCs are non-spiking for most of the maturation process, and inhibitory and excitatory synaptic events are detected before voltage-dependent Na^+^ currents [131]. This developmental pattern is the inverse of that observed in prenatally generated GCs, where AP generation precedes synaptic activity, and may reflect their integration into OB circuits that are already functional [131]. Adult-born GCs exhibit odor-specific responses and respond most strongly to novel odors soon after they have completed differentiation and synaptic integration into OB circuits, when they are about three weeks old [221]. Indeed, at the population level, they respond more strongly to novel odors than mature pre-existing GCs, suggesting a distinct role in odor processing [221].

Adult-born GCs also respond to odors soon after their arrival in the OB: as early as three days after arriving in the OB, some newborn GCs had well-elaborated dendritic trees, and their dendrites in the EPL responded to odor stimulation [222]. The same study tracked odor responses in identified GC dendrites over time using 2-photon calcium imaging, finding that young adult-born GCs exhibit larger responses and are broadly tuned to odors. After three further weeks of maturation, most adult-born GCs became narrowly tuned to odors, but a subset underwent broadening of odor tuning, likely reflecting different development profiles for distinct GC subtypes. Interestingly, odor enrichment prolonged the time period during which larger, broadly tuned responses were seen, suggesting that refinement of adult-born GC connectivity is activity dependent.

A unique feature of GC neurogenesis is that the laminar position of a GC soma is influenced by neuronal birth date: GCs generated prenatally and before P7 preferentially occupy the superficial GCL, whereas those generated at P14 and later are more likely to occupy the deeper regions of the GCL [64,223]. Interestingly, GCs in the MCL and superficial GCL differ in morphology from those in the deep GCL; of particular interest, dendrites of superficial GCs project predominantly within the superficial EPL, while dendrites of deep GCs are mainly in the deep EPL [224,225,226]. This means that superficial GCs should predominantly contact TC lateral dendrites while deep GCs predominantly contact MC dendrites [225,226]. In terms of functional differences, superficial GCs are more likely than deep GCs to fire action potentials following stimulation of a single glomerulus, and superficial GCs exhibit suprathreshold responses while deep GCs typically exhibit subthreshold responses following odor stimulation in vivo [224,227]. This leads to the hypothesis that GCs that are born early (embryonically or before P7) preferentially receive excitation from and subsequently inhibit TCs while later born GCs are more likely to be excited by and inhibit MCs. If true, then adult-born GCs could play quite a distinct role to their earlier born counterparts by engaging preferentially with a distinct OB output pathway.

### 4.2. Survival of Postnatally Generated GCs

There has been considerable controversy concerning the survival rate of postnatally generated GCs, as well as differing conclusions as to whether adult GC neurogenesis results in addition or replacement. Several early studies that employed BrdU or ^3^H-thymidine labeling found that the density of postnatally born GCs in the OB peaked about two weeks after their birth, but 25–50% of these neurons were then eliminated between 15 and 45 days of neuronal age [53,219,228,229,230]. Notably, neonatally generated superficial GCs survived for longer than their adult-born counterparts [64]. Corroborating this, a study that employed a Cre-mediated strategy to label postnatally born GCs found that nearly all deep GCs are replaced by new neurons over a 12-month period, whereas only ~50% of superficial GCs are replaced during this time [231].

In contrast, another study that employed retroviral labeling found virtually no cell death of 28-day-old postnatally born GCs [232]. Similarly, a more recent study that employed chronic in vivo 2-photon imaging found minimal cell death of adult-born GCs, suggesting that there is net addition of GCs in the adult OB [233]. Additional experiments in this study suggested that the discrepancy amongst studies of GC survival could be due to thymidine analog toxicity: they found that commonly used concentrations of BrdU and EdU resulted in substantial cell death of adult-born OB neurons. This is a key point to consider, and we note that not only the single injection dose (typically ranging from 5 to 300 mg/kg), but also the total dose and administration time period, with protocols typically comprising between one and ten injections across one to seven days, may impact thymidine analog toxicity. However, not all studies showing dramatic loss of postnatally born GCs employed thymidine analogs [231]. Furthermore, a genetic fate mapping strategy suggested that GC neurogenesis switches from addition to replacement beyond P35, as new neurons continue to be generated but the total number of GCs does not increase significantly [223]. Another important factor to consider is the depth of the GCs being studied, with multiple techniques highlighting greater survival rates of superficial GCs [64,223,231], which are much more accessible for in vivo imaging. Mouse age, strain and housing conditions may also contribute, and the labeling strategies that did not employ thymidine analogs also vary widely, although no combination of the above factors provides a satisfying explanation for the full range of data on postnatal-born GC survival. Future studies should seek to resolve these discrepancies.

Another important question has been whether GC survival is activity dependent, as this could provide a mechanism for experience-dependent modification of OB circuitry. Suppressing intrinsic activity of postnatally generated GCs decreased their survival, whereas artificially increasing their activity protected GCs from cell death [232]. Importantly, these activity manipulations did not affect synaptogenesis, and increased survival did not rely on a specific pattern of activity, indicating that the overall level of intrinsic activity is key to regulating postnatally born GC survival [232]. OSN axotomy increased neuroblast apoptosis in the RMS, suggesting that OB input is important for survival of GC precursors, although interestingly, this was counterbalanced by increased SVZ and OB core neurogenesis [66]. Naris occlusion also reduced GC survival during a critical period 14–45 days after neuronal birth [228,234,235]. Importantly, this effect on survival was evident only in the deep half of the GCL, indicating that it is GC subtype specific [234]. Survival may be related to synaptic integration into OB circuits, as naris occlusion during synaptic maturation of adult-born GCs resulted in a loss of spines in their distal apical and basal dendrites that was rescued by increasing their excitability [236]. As seen for loss of sensory input, OCNC1 knockout mice, which are anosmic due to disruption of OSN signal transduction, exhibited increased GC death 15–45 days after their generation [219].

Whether odor exposure affects postnatal born GC survival depends on odor complexity, pattern and/or duration of exposure. Three weeks of continuous exposure to a complex mixture of 20 odors significantly increased survival without affecting proliferation [237]. Importantly, this long-term odor exposure also improved short-term memory of novel odors that mice had not previously been exposed to [237]. In contrast, four weeks of intermittent odor exposure (“odor familiarization”) to a mixture of three food odors did not affect newborn GC survival but did increase their population response to these odors, whereas the population response of pre-existing GCs was reduced [221]. Mouse age may also be an important factor: an odor enrichment paradigm consisting of all-day exposure to a rotating set of 15 odors for 34 days increased GC survival in 2-month-old, but not 10-month-old, mice [216]. In agreement with this, enrichment with a single odor increased the number of GCs generated in early postnatal life [64].

### 4.3. Functional Role of Postnatally Generated GCs

The activity dependence of GC survival raises the interesting possibility that GC neurogenesis provides a flexible substrate that enables continuous optimization of OB circuits to salient odor information. Hence, numerous studies have investigated the role of postnatally born GCs in olfactory learning, and indeed, multiple forms of olfactory learning have been found to modulate survival of postnatally generated GCs, as detailed below. Overall, the studies that we will discuss support a key role for postnatally born GCs in certain forms of olfactory learning and memory. However, there are two important caveats to consider in interpreting these studies. First, variations in experimental design complicate their interpretation and comparison. Second, experimental manipulations designed to block GC neurogenesis will also prevent JGN neurogenesis. Similarly, optogenetic manipulations of adult-born OB neurons may modulate adult-born JGNs in addition to GCs, depending on whether a surface LED or implanted fiberoptic is used. While adult-born GCs vastly outnumber adult-born JGNs, and most of the studies below also present other lines of evidence supporting a role for adult-born GCs in particular learning and memory tasks, a potential contribution of adult-born JGNs to olfactory learning and memory should also be carefully considered. An emerging role for adult-born GCs in other olfactory-guided behaviors is also discussed.

#### 4.3.1. The Role of Postnatally Generated GCs in Olfactory Perceptual Learning

Non-associative olfactory perceptual learning, a form of implicit learning resulting from odor exposure, was accompanied by increased survival of newborn GCs and was prevented by cytosine arabinose (AraC)-mediated ablation of adult-born GCs either before or during odor exposure [238]. There was a similar increase in adult-born GC density for simple (one or two odor pairs) and complex (3–6 odor pairs) olfactory perceptual learning tasks, and optogenetic silencing of adult-born GCs prevented discrimination irrespective of the complexity of the perceptual learning task [239]. However, activation of adult-born GCs by the exposed odor was increased only for more complex tasks, suggesting greater recruitment of adult-born neurons. Importantly, successive perceptual discrimination tasks were supported by separate populations of adult-born GCs, with the neurons that were rescued from cell death persisting for as long as mice remembered the task [240]. However, learning a second task within three weeks of the first resulted in retrograde interference and reduced survival of the adult-born GCs recruited by the first task, unless those odors remained present in the environment. Hence, adult-born neurons can regulate memory persistence dependent on its ongoing relevance. Mechanistically, the increase in adult-born GC density due to perceptual learning increased inhibition onto MCs and TCs, likely enhancing learning by leading to sparser and less overlapping odor representations [241]. Furthermore, the role of adult-born neurons in perceptual learning is associated with increased GC dendritic spine density. 12-month-old mice exhibit impaired olfactory perceptual learning, and while adult-born GCs continue to be incorporated, they no longer exhibit a learning-induced increase in basal dendrite spine density [242]. Interestingly, for simple tasks, spine density increased only on the basal and distal apical dendrites, whereas more complex tasks also increased spine density on the proximal apical dendrites [239].

#### 4.3.2. The Role of Postnatally Generated GCs in Olfactory Associative Learning

Two early studies that employed associative olfactory discrimination learning generated somewhat conflicting results. One study found a selective learning-related reduction in cell death of three-week-old GCs, which was not seen when the reward was not paired to one of the two tested odors [229]. Surprisingly though, this increase in survival was specific to areas activated by the unrewarded odor [229]. In the other study, a different learning paradigm reduced the survival of 45-day-old GCs, with some of the surviving GCs being activated by the rewarded odor [243]. These surviving GCs were rescued from apoptosis by learning [244]. Subsequent studies suggest that the key difference in survival outcome may be neuronal age. Mature five-week-old GCs were preferentially recruited by associative olfactory learning, whereas immature two-week-old GCs were more strongly activated by passive odor stimulation [245]. Associative olfactory discrimination learning using a go/no-go paradigm enhanced survival of new immature GCs but promoted elimination of more mature GCs, while leaving both very young GCs that were just reaching the OB and fully mature GCs unaffected [246]. Importantly, both positive and negative effects of learning on GC survival were specific to deep GCs; while some superficial GCs were generated, learning did not modulate their survival [246]. Hence, learning can bidirectionally modulate survival of a subset of GCs at specific stages of their maturation.

Multiple studies then asked whether ablation of postnatally born GCs, using several different approaches, affected associative olfactory learning, generating results that remain somewhat contradictory. AraC treatment, which ablates newborn neurons by preventing DNA synthesis, affected short-term but not long-term olfactory memory [247], while a genetic strategy that selectively ablated newborn neurons did not affect olfactory memory [231]. In contrast, SVZ irradiation had a negative effect on long-term but not short-term olfactory memory, suggesting that continuous production of GCs may play a specific role in memory consolidation [248]. SVZ infusion of an anti-mitotic drug during task learning also resulted in significant deficits in long-term olfactory memory [249]. Furthermore, the newborn GCs involved in task retention were activated by the rewarded odor. Finally, genetic ablation of adult-born GCs impaired fine but not coarse odor discrimination learning using a two-alternative choice task and demonstrated an important role for adult-born GCs in enhancing MC pattern separation during task engagement [250]. A potential explanation for these discrepancies was suggested to be the type of olfactory associative learning paradigm employed. Non-operant conditioning does not affect long-term memory [231,247], likely because it does not alter newborn GC survival [251]. In contrast, operant learning, which affects newborn GC survival [244,248,249,252], is reliant on ongoing GC neurogenesis [248,249,250,251]. However, deficits in non-operant olfactory associative learning following genetic ablation of recently born GCs were then reported [253]. By controlling the timing of ablation, this study showed that memory expression was only impaired if the ablated GCs were at least 10 days old, and that their role was time limited, as ablation a month after training did not result in amnesia. Importantly, the role of adult-born GCs was similar in juvenile and adult mice, suggesting that the age of the GC is a key factor for its role in olfactory learning and memory.

Many other studies, employing a range of experimental strategies, collectively support an important role for postnatally born GCs in olfactory learning and memory. Erasure of a previously learnt odor-reward association suppressed the survival of GCs that had been preserved by task learning [254]. Furthermore, preventing caspase-mediated apoptosis during extinction, which preserved adult-born GCs, also prevented erasure of the odor-reward association. Optogenetic stimulation of adult-born neurons (GCs and JGNs) also accelerated learning of a difficult odor discrimination task and improved task memory, likely due to enhanced GABAergic inhibition of OB principal neurons, and enhanced value updating during reversal learning [255,256]. While adult-born GCs were not required to learn a simple odor discrimination task, they were required for reversal learning, suggesting a key role in flexible olfactory associative learning [223]. Similarly, increasing adult neurogenesis above its normal setpoint by manipulating expression of cell cycle regulators enhanced learning of a difficult odor discrimination task but not an easier one [257]. Therefore, it appears that postnatally born GCs are necessary for difficult odor discrimination learning, whereas pre-existing GCs can compensate during easier tasks.

#### 4.3.3. Cellular and Circuit Mechanisms Underlying Enhanced Olfactory Associative Learning

A plethora of cellular and circuit mechanisms have been proposed to explain how postnatally generated GCs enhance olfactory associative learning. In terms of structural plasticity, go/no-go operant learning increased spine density specifically on basal dendrites of adult-born GCs, which receive excitatory feedback from olfactory cortex [258]. Indeed, learning strengthened excitatory olfactory cortical inputs to adult-born GCs, providing evidence for input-specific plasticity during learning. In contrast, operant learning of an odor-reward association task reduced dendritic spine density on apical dendrites of adult-born GCs, and reduced inhibition onto MCs and TCs [241]. The different learning tasks employed in these two studies may explain the differing results. Adult-born GCs, but not early born GCs, also exhibit a unique form of plasticity that may enable rapid adaptation to a changing odor environment: principal neuron activity induces formation of spine head filopodia, which results in activity-dependent spine relocation [259]. There is also evidence that GABAergic input to adult-born GCs from the basal forebrain, which emerges as they enter the OB and strengthens as they integrate, promotes their survival [260]. Two-week-old GCs also exhibited long-term potentiation (LTP) at a subset of their glutamatergic inputs, which faded as they matured, providing a potential substrate for neurogenesis-dependent olfactory learning [208]. Finally, survival of adult-born GCs during learning is protein synthesis-dependent, i.e., it requires memory consolidation to occur [252].

At the circuit level, adult-born GCs play an important role in shaping odor representation by MCs and TCs. Adult-born GCs make an important contribution to recurrent and lateral inhibition of MCs and TCs, as well as in synchronizing principal neuron activity: their ablation reduces the frequency of odor-evoked OB gamma oscillations [247]. Furthermore, adult-born neurons have been shown to enhance MC pattern separation during engagement in odor discrimination tasks [250]. When adult-born neurons were genetically ablated, MC representations of similar odorants were more ambiguous, which was primarily due to a reduction in suppressive odor responses. Interestingly, young adult-born GCs specifically alter MC odor coding by sharpening their odor tuning and improving their ability to discriminate odors [261]. These effects are lost as adult-born GCs matured and are likely due to the greater excitability and broad input connectivity of young GCs.

#### 4.3.4. Other Functions of Postnatal GC Neurogenesis

While there has been a strong focus on the role of postnatal GC neurogenesis in learning and memory, evidence for other important functions of adult-born GCs has emerged more recently. Chronic unpredictable mild stress, which led to anxiety-like behaviors and anhedonia, also altered odor hedonics by reducing the investigation of pleasant odors. Interestingly, this was accompanied by a reduced density of newborn GCs, which could be due to reduced GC neurogenesis and/or survival during the stress paradigm [262]. Furthermore, the stress paradigm selectively decreased the density of adult-born GCs that responded to pleasant odors, while responses to unpleasant odors were unchanged. These changes in adult-born GC density and pleasant odor responsiveness may therefore underlie the stress-related alteration in odor hedonics.

Pregnancy also impacts the generation and integration of adult-born GCs. In lactating mothers (3–4 days after birth), the density and turnover of dendritic spines were reduced only for adult-born GCs, while the density of presynaptic terminals in their dendrites was increased [263]. These changes suggest enhanced integration of adult-born GCs that were generated during pregnancy into the OB network, which may modulate OB principal neuron activity to mediate maternal behaviors. More recently, it has been shown that pregnancy triggers the generation of transient waves of short-lived GCs, which unusually for adult-born GCs, are localized predominantly to the MCL and superficial GCL [264]. Through pregnancy, stem cells in distinct subregions of the SVZ, including some that are normally quiescent, generate these pregnancy-associated GCs. GCs destined for the MCL were generated during very early gestation (gestational day 2.5 or earlier), while GCs that incorporated into the superficial GCL were generated later (day 4.5–7.5). The density of neuropeptide Y-expressing GCs (a very understudied subpopulation) increased significantly in both the MCL and superficial GCL, while CR+ GC density was also elevated in the superficial GCL. Most importantly, pregnancy-associated GCs had clear physiological relevance. They disappeared within days when pups were prematurely removed from the nest, and pregnancy-associated MCL GCs play an important role in own pup odor detection, while pregnancy-associated superficial GCL neurons enhance preference for pups over other objects.

### 4.4. Postnatal Neurogenesis of Neurochemically Defined GC Subtypes

#### 4.4.1. CaMKII-Expressing GCs

Several different GC subtypes have also been defined using neurochemical markers, which exhibit partially overlapping expression. In the OB, CaMKIIα is expressed only in GCs [265]. CaMKIIα-expressing GCs account for 50% of the MCL and GCL populations and are distributed throughout the superficial and deep GCL [211,265,266]. CaMKIIα expression defines a functionally distinct population of GCs that receives weaker inhibitory input than other GCs and hence is preferentially activated by odor input [266]. CaMKIIα+ GCs are required for odor discrimination in a habituation/dishabituation assay and discrimination of complex odor mixtures in a Go/No-go task, but not for olfactory perceptual learning [266]. CaMKIIα mRNA and protein are localized to GC dendrites in an activity-dependent manner, and removal of the 3′ UTR, which is required for dendritic localization, causes impaired olfactory associative learning [267]. CaMKIIα+ GCs continue to be generated at a high level postnatally: half of GCs generated across ages spanning from P12 to P56 express CaMKIIα [266]. However, whether these postnatally born CaMKIIα+ GCs play roles that are distinct from earlier-generated CaMKIIα+ GCs is unknown.

#### 4.4.2. Calretinin-Expressing GCs

CR-expressing (CR+) GCs are preferentially localized in the superficial GCL and are morphologically similar to other surrounding GCs [268]. They receive similar excitatory inputs but fewer inhibitory inputs on primary dendrites than do CR- GCs in the superficial GCL [268]. CR+ GCs are preferentially activated during odor dishabituation (i.e., discrimination) compared to CR- superficial GCs and contribute to fine odor discrimination learning using similar enantiomer mixtures in a Go/No-go assay [268]. CR+ GC neurogenesis peaks around birth but continues at high levels into adulthood [147,268]. Early born (P10–12) and adult-born (2–4 month) CR+ GCs are morphologically indistinguishable and receive similar numbers of inhibitory inputs [268]. However, there has been no direct test of whether there are differences in the function of CR+ GCs born at different times. It is important to note though that CR+ GCs are superficially located yet continue to be generated into adulthood. This indicates, in agreement with previous studies [64,223], that laminar position is not an absolute predictor of GC birth date, and therefore that at least some adult-born GCs are likely to inhibit TCs rather than MCs.

#### 4.4.3. 5T4-Expressing GCs

5T4 is expressed in a subset of GCs located in the MCL, IPL and very superficial GCL [269,270]. Their dendrites ramify predominantly in the superficial EPL [271]. 5T4+ GCs are generated 5T4 expression is downregulated by loss of sensory input, with 5T4 overexpression rescuing the activity-dependent reduction in dendritic branching [269]. In 5T4 knockout mice in which the coding region is replaced with the lacZ gene, lacZ+ GCs exhibited reduced dendritic branching and received fewer excitatory inputs [269,270]. Radial migration in the OB was also affected, with some lacZ+ GCs being abnormally located in the deep GCL [269]. At the behavioral level, both 5T4 KO mice and OB-specific 5T4 knockdown mice had higher odor detection thresholds and showed impaired odor discrimination learning in a reward association task [270]. While it is clear that 5T4+ GCs are generated mainly during the embryonic and neonatal stages [147,270], one study found significant ongoing 5T4+ GC neurogenesis in P10-P30 mice [147] while another found very little neurogenesis in 2–8-week-old mice [270]. Hence, it remains unclear what contribution postnatally generated 5T4 GCs make to olfactory processing.

#### 4.4.4. mGluR2-Expressing GCs

mGluR2+ GCs account for approximately a third of GCs and are present throughout the GCL and MCL [272,273]. There is significant overlap of mGluR2 and CR expression: approximately 70% of CR+ GCs are mGluR2+, whereas CR+ accounted for only 25% of mGluR2+ GCs [273]. Conversely, less than 10% of 5T4+ GCs expressed mGluR2, and 5T4+ GCs accounted for over a third of mGluR2- GCs [273]. Accordingly, the density of CR+ GCs was significantly lower after immunotoxin-mediated mGluR2+ GC ablation but recovered substantially within 8 weeks, whereas 5T4+ GC density was unaffected [273]. mGluR2+ GCs are generated both in early postnatal and young adult (8–12-week-old) mice, and their incorporation is upregulated after selective immunotoxin-mediated ablation in the dorsal OB, with these new mGluR2+ GCs having larger spines [273]. The persistence of mGluR2+ GC neurogenesis beyond 12 weeks has not been studied. Furthermore, the specific functional roles of mGluR2+ GCs, aside from those established for the CR+ subset, remain to be elucidated.

## 5. Conclusions and Future Directions

Postnatal neurogenesis plays diverse roles in the olfactory system that impact processes ranging from homeostasis to learning and memory. While OSN neurogenesis is necessary to maintain sensory input to the OB, the complement of OSNs expressing different ORs is continuously optimized by activity and experience, enabling ongoing adaptation of odor input to the brain to match current environmental conditions. It is also clear that even after large-scale ablation, OSNs can repopulate effectively within a few weeks. Nevertheless, regeneration of OSN axonal projections is error-prone, with regenerated glomerular maps potentially altering odor coding. Hence, it will be important to determine how accurately the reinnervation of glomeruli by regenerated OSNs can be promoted to completely restore olfactory function after widespread loss of OSNs. Furthermore, what role, if any, is played by recently born OSNs in learning and memory remains unclear [274,275], leaving this as another important future direction.

Given that relatively small numbers are generated postnatally compared to GCs, JGNs have received less attention, leaving the role played by their ongoing neurogenesis rather unclear. However, their location in the input layer of the OB positions neurogenesis here to play an important role in modulating odor processing and plasticity. The best-studied subtype, GL DA neurons, provide some interesting principles, the generalizability of which will need to be determined in future studies. First, adult neurogenesis increases the size of the DA neuron population, rather than just replacing lost neurons. This provides a mechanism for the ongoing experience-dependent sculpting of GL circuits and suggests that adult SVZ neurogenesis can add functionality. Second, SVZ neurogenesis enables the repopulation of DA neurons after cell death induced by a loss of sensory experience, suggesting that SVZ neural stem cells are an important reservoir for neural circuit repair, a topic that has received little attention in the context of GCs.

Postnatal GC neurogenesis stands out as a key contributor to olfactory learning and memory. In contrast, it is interesting to note that unlike for GCs, perceptual learning did not enhance GL neuron survival [238,246]. Adult-born GCs are important for both perceptual and some forms of associative learning; indeed, they are necessary for learning of certain tasks. However, there remain several discrepancies between studies that complicate the interpretation of the roles of postnatal GC neurogenesis. These may be resolved by a greater focus on the roles of individual GC subtypes in particular tasks. Given the broad and overlapping expression of established GC markers, it will be important both to determine whether there are additional GC markers that define smaller subclasses, and to apply intersectional genetic strategies to label and manipulate specific GC subsets, e.g., mGluR2+CR+ vs. mGluR2+CR-.

There are also some important overarching themes that emerge from studies of both OE and SVZ neurogenesis. First, immature neurons are not merely precursors: they exhibit heightened plasticity and can play distinct roles in olfactory processing while they are integrating into OB circuits. Second, bidirectional modulation of newborn neuron survival by activity was prevalent across multiple neuronal types. In many cases, increases vs. decreases in activity, whether through manipulations of intrinsic activity or sensory input, had opposing effects on survival, highlighting the capacity of the olfactory system to adapt to changes in the odor environment. Under natural conditions, where novel odors are frequently encountered, this capacity may be important for organismal survival. Third, a major question posed regarding adult neurogenesis is whether it serves simply as a mechanism for replacement or can add new functions. In the olfactory system, while it clearly adds new functionality, it is notable that replacement itself can alter the properties of the circuit by generating new neurons that are tuned to the experience of the animal. Finally, the relationship between OE and SVZ neurogenesis is very underexplored. If the connectivity patterns established overall for inhibitory JGN subpopulations [153,154,155,173] also hold for adult-born JGNs, then it is likely that some postnatally generated dopaminergic neurons, but not CR+ or CB+ PGCs, receive direct input from newborn (and potentially immature) OSNs. Furthermore, whether or not and how the modulation of neurogenesis in one olfactory stem cell niche regulates the other, and how the plasticity associated with continuous incorporation of newborn OSNs impacts newborn OB interneurons, vice versa, are also important unanswered questions. Overall, a wealth of studies collectively demonstrate the key role of postnatal neurogenesis in maintaining olfactory function, promoting olfactory learning, and regenerating damaged circuits.
